# Sustainable food system policies need to address environmental pressures and impacts: The example of water use and water stress

**DOI:** 10.1016/j.scitotenv.2020.139151

**Published:** 2020-08-15

**Authors:** Davy Vanham, Adrian Leip

**Affiliations:** European Commission, Joint Research Centre (JRC), Ispra, Italy

**Keywords:** Sustainable, Food system, EU, Policy, Water use, Water stress

## Abstract

Sustainable food systems are high on the political and research agendas. One of the three pillars of sustainability is environmental sustainability. We argue that, when defining related policies, such as policies under the European Green Deal, both environmental pressures and impacts carry important and complementary information and should be used in combination. Although the environmental focus of a sustainable food system is to have a positive or neutral impact on the natural environment, addressing pressures is necessary to achieve this goal. We show this by means of the pressure water use (or water footprint) and its related impact water stress, by means of different arguments: 1) Water use and water stress are only weakly correlated; 2) water use can be evaluated towards a benchmark, addressing resource efficiency; 3) water use is used for resource allocation assessments within or between economic sectors; 4) water amounts are needed to set fair share amounts for citizens, regions, countries or on a global level 5) the pressure water use requires less data, whereas water stress assessments have more uncertainty and 6) both provide strong communication tools to citizens, including for food packaging labelling. As a result, we present a water quantity sustainability scheme, that addresses both water use and water stress, and can be used in support of food system policies, including food package labelling.

## Highlights

• The European Green Deal includes a Sustainable Food system (SFS) policy.
• SFS policies need to address environmental pressures and impacts.
• We show this with the example water use (pressure) and water stress (impact).
• We present a water quantity sustainability scheme.
• This scheme can be used for SFS policies, including food package labelling.

## Graphical abstract

**Figure:**
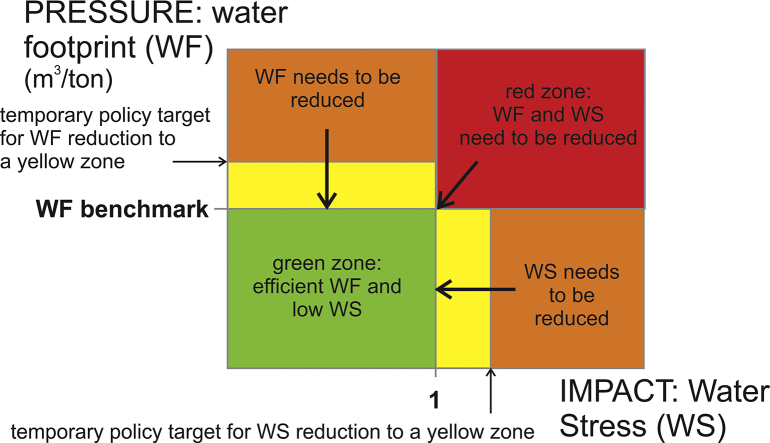

## 1 Introduction

Both in academia and in policy-making, it has now been recognized that sustainable food systems (SFSs) need to be assessed in an integrated manner, in order to grasp their complexities. Tools to do so include the SDG framework or the water-energy-food (WEF or FEW) nexus (Vanham et al., 2019a). SFS analyses require interdisciplinary research, breaking knowledge and policy-making silos. Recent research regarding global sustainable food systems (SFSs) has made substantial progress on integrating human health and environmental sustainability (Gerten et al., 2020; Springmann et al., 2018; Willett et al., 2019).

Latter studies use a selection of pressure indicators and relate them to their respective planetary boundaries to evaluate the environmental sustainability of the food system. Environmental pressure indicators are different from impact indicators, as they inform users on the pressure human activities place on ecosystems (e.g. water use, greenhouse gas emissions, losses of reactive nitrogen) (Holmatov et al., 2019; Leip et al., 2014; Pahlow et al., 2015). They quantify either resource use or pollution, or both. These pressures are the cause of environmental impacts such as water stress, coastal and marine eutrophication, climate change or air pollution, to name few examples of impacts(Bouraoui and Grizzetti, 2011; Leip et al., 2015; Malagó et al., 2019; Mekonnen and Hoekstra, 2016).

Environmental footprints are pressure indicators that track and integrate pressure along a supply chain. The greenhouse gas footprint (also called carbon footprint) measures pollution, the water and land footprints measure resource use and the nitrogen and phosphorus footprints measure both (Kastner et al., 2012; Leach et al., 2012; Sutton et al., 2011; Vanham et al., 2019a). To evaluate the relevance measured by these footprints, they have to be related to their respective local and/or planetary boundary. For the carbon footprint, this is a global boundary. For the four other footprints, these are regional or local boundaries (Carpenter and Bennett, 2011; de Vries et al., 2013; Hoekstra, 2016; Hogeboom et al., 2020; Steffen et al., 2015; Vanham et al., 2019a).

SFSs are high on the policy agenda's. The European Green Deal includes a new Farm to Fork Strategy on sustainable food along the whole value chain, which aims at designing a fair, healthy and environmentally-friendly food system (EC, 2019). More specifically, the strategy will set out how to (EC, 2020):

• Ensure sustainable primary production;
• Stimulate sustainable food processing, retail, hospitality and food services' practices;
• Promote sustainable food consumption, facilitating the shift towards healthy, sustainable diets;
• Reduce food loss and waste.

The recently published joint FAO and WHO report “Sustainable healthy diets – guiding principles” (FAO and WHO, 2019), which includes the three pillars of sustainability (social, economic and environmental), defines that “*sustainable healthy diets are dietary patterns that promote all dimensions of individuals' health and wellbeing; have low environmental pressure and impact; are accessible, affordable, safe and equitable; and are culturally acceptable*”. This document, launched on World Food Day 2019, aims to support the efforts of countries as they work to transform food systems to deliver on sustainable healthy diets. It explicitly refers to environmental pressures and impacts.

Although the environmental focus of a SFS is to have a positive or neutral impact on the natural environment (FAO, 2018), addressing pressures is necessary to achieve this goal. The aim of our paper is to clarify why addressing both pressures and impacts are relevant for policy-making, using the example of the pressure water use and impact water stress. We illustrate this using published data. We conduct a new assessment of the global water footprint and related water stress for irrigated maize as exemplary case study. Latter assessment uses the same approach and datasets as a recent global water stress assessment done for groundnuts and treenuts (Vanham et al., 2020). We show that for defining SFS policies, an accounting framework based on both pressures and impacts is needed, to ensure an adequate pathway is chosen to reach SFSs.

## 2 Methodology

As pressure indicator, we use the consumptive water footprint (WF) as defined by Hoekstra and Mekonnen (2012). This indicator comprises a blue and green WF component. Blue water refers to water in rivers, lakes and aquifers. Green water is the soil water held in the unsaturated zone, formed by precipitation and available to plants (Falkenmark et al., 2019). Irrigated agriculture receives blue water (from irrigation) as well as green water (from precipitation), while rainfed agriculture receives only green water. We do not account for the grey WF component. We thus only assess an environmental pressure that measures resource use, not pollution.

We differentiate between the WF of production and the WF of consumption. The first refers to the WF of a product or in the case of a geographical region, to the sum of the direct and indirect water use of domestic freshwater resources of that region. The latter refers to consumption at the end of the supply chain, i.e. the total volume of freshwater used to produce the goods consumed by a citizen, or by the inhabitants of a geographical region.

We take global maize production as a case study for our assessment, for which we use WF of production and irrigated yield data of Mekonnen and Hoekstra (2011). These are global gridded data at a resolution of 5 by 5 arc minute (or 10 by 10 km at the equator). We relate the blue WF of production of maize to global water stress. Water stress (WS) relates water use to water availability (Vanham et al., 2018b). Latter authors identified seven key elements that need to be considered for a water stress indicator, of which one is groundwater. The paper also evaluates whether SDG indicator 6.4.2 considers these elements. To account for WS, we use the spatially distributed WS data of Mekonnen and Hoekstra (2016). This indicator puts blue WF amounts (the accounting phase in a Water Footprint Assessment) in relationship to local blue water availability (the impact assessment phase) (Hoekstra et al., 2011). Blue WS in Mekonnen and Hoekstra (2016) is computed as:

$$\text{Blue}\;\mathrm{WS}=\frac{\text{blue water footprint}}{\left(\mathrm{WA}-\mathrm{EFR}\right)}$$

with WA = total blue water availability, and EFR = environmental flow requirements.

Following blue WS thresholds are used: values until 1 (low blue WS), 1–1.5 (moderate blue WS), 1.5–2 (significant blue WS) and more than 2 (severe blue WS).

We do not address green water scarcity (Schyns et al., 2019). Green water scarcity refers to the competition over limited green water flows. Different definitions exist (Schyns et al., 2015), and although the analysis of Schyns et al. (2019) provides a significant advancement in the analysis and data availability on the topic, the consensus on how to compute blue water stress is much more profound than on green water scarcity. We expect advancement on the latter in the near future, but choose not to include it here.

We briefly address WF benchmarks (Mekonnen and Hoekstra, 2014) of irrigated maize (expressed as m^3^/ton or l/kg) indicating efficient water use. The WF is the inverse of water productivity (WP, expressed as ton/m^3^ or kg/l). Other definitions of water productivity do exist, such as amount of product per water withdrawn. Within the SDG framework, SDG indicator 6.4.1 measures water use efficiency as value added per water withdrawn (Vanham et al., 2018b). We focus only on WF benchmarks.

## 3 Results and discussion

### 3.1 Water use and water stress are only weakly correlated

**Fig. 1:**
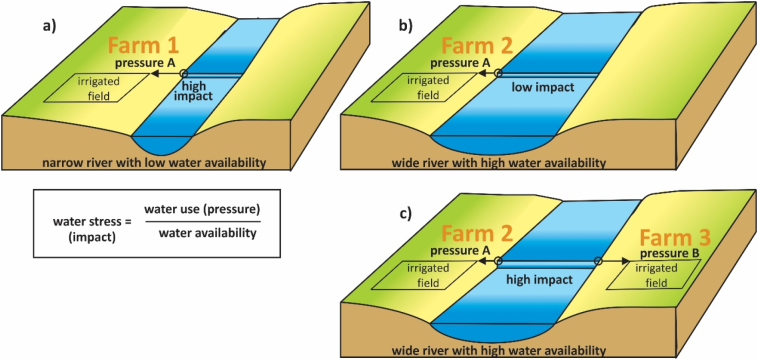
Selected different situations how a farm with a certain amount of irrigation water use (pressure) from a river, can have different impacts (water stress). Many more are possible, as shown in Fig. 2. We use here a specific situation of farm 1 and 2 that use the same amount of irrigation water (pressure A) from a river. Farm 3 uses a different amount (pressure B). a) Farm 1 abstracts water from a river with low water availability, resulting in a high impact. b) Farm 2 abstracts the same amount of water from a river with high water availability, resulting in a low impact. When only focusing on impact, farm 1 seems to be unsustainable, farm 2 sustainable. c) When farm 3 additionally uses pressure B at the same location, the impact of farm 2 in that location changes from low to high. We assume in all situations for simplicity that all abstracted water is consumed (no return flow).

Regarding water stress, there are different reasons why a certain water use (pressure) can have different impacts. This includes a situation with different water availability within a location, or a situation where in the same location other users exert an additional pressure. As conceptually presented in Fig. 1a and b, depending on a specific local water availability, an identical water use (pressure A) can exert high (farm 1) to low (farm 2) water stress. Fig. 1b and c show that, depending whether there are other water users (such as pressure B from farm 3) or not in a certain location, a certain pressure A (farm 2) can have a low (Fig. 1b) or high (Fig. 1c) impact.

**Fig. 2:**
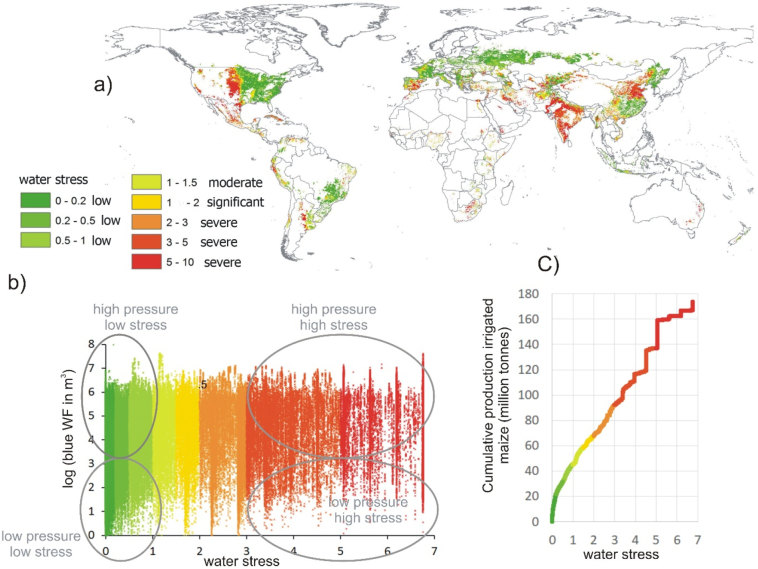
Irrigated maize produced under different levels of average annual water stress, a) global WS map for irrigated maize (where annual production per pixel exceeds 1 ton); b) graph showing the relation between the WS value (X-axis) and the blue WF of production of maize (in m^3^/year)(Y-axis) for all pixels; c) the cumulative production (million tonnes) of irrigated maize under different levels of average annual water stress. (For interpretation of the references to colour in this figure legend, the reader is referred to the web version of this article.)

Fig. 2 shows a geographically detailed analysis of irrigated maize produced under different levels of average annual WS. The total global blue WF of production of maize amounts to 51 km^3^/yr (Mekonnen and Hoekstra, 2011). Computing the relation between water use (the blue WF) with WS, displays a very heterogeneous data scatter plot. Low pressures can relate to low to high impact. High pressures can relate to low to high impacts. The correlation between both datasets is with an amount of 0.16 a weak positive correlation. Only addressing one of the two indicators thus not automatically captures the other.

Overall 74% of irrigated maize is produced under WS (Fig. 2c). Weighted according to production, the average WS amount of irrigated maize amounts to 2.8.

### 3.2 Water use can be evaluated towards a benchmark, addressing resource efficiency

From an impact point of view, pressures with low impact seem to have no priority to address. However, different global modelling studies have shown that, to keep within planetary boundaries, the food system needs to address resource efficiency and dietary shifts (Gerten et al., 2020; Springmann et al., 2018; Willett et al., 2019). The former includes sustainable intensification (Garnett et al., 2013) and the closure of yield gaps (Mueller et al., 2012), thus optimising resource use pressures such as water, land, nitrogen or phosphorus use and reducing pollution pressures such as greenhouse gas emissions and nitrogen pollution. In water footprint jargon, this means attaining water footprint benchmarks (Mekonnen and Hoekstra, 2014).

**Fig. 3:**
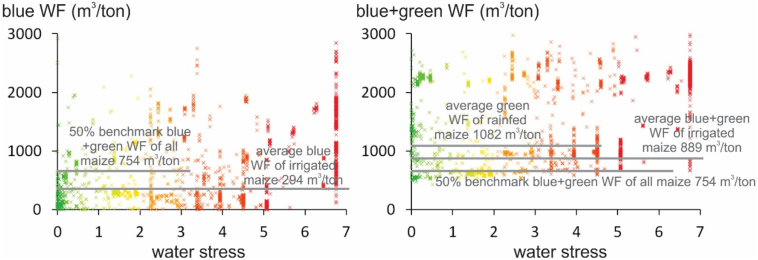
Unit WF amounts (m^3^/ton) for a random sample of irrigated maize grid cells, (left) for the blue WF and (right) the blue+green WF. The green+blue WF of all maize (irrigated and rainfed) at the 50th production percentile is shown (Mekonnen and Hoekstra, 2014). (For interpretation of the references to colour in this figure legend, the reader is referred to the web version of this article.)

Fig. 3 shows a random selection of unit WF amounts for irrigated maize, spanning all continents over the world, for both the blue WF separately as well as the blue+green WF. The green+blue WF of all maize (irrigated and rainfed) at the 50th production percentile is 754 m^3^/ton, which can be set as benchmark. Regarding the blue WF, the figure shows that for low to high water stress, irrigated maize WF amounts are both below and above the benchmark value. Also for the green+blue WF, the same observations are made. In other words, a targeting of lowering WF amounts exceeding benchmarks only in moderate to high water stress regions, neglects the fact that also in low water stress regions WFs should be reduced to benchmark amounts. Also the WF of rainfed maize should be reduced to benchmark amounts, in order to alleviate water stress in other regions. Different strategies to reduce WFs to benchmarks are listed by Mekonnen and Hoekstra (2014).

### 3.3 Alleviating water stress needs allocating water use within and between economic sectors

The aim of a SFS is to achieve a water stress up to one. Part of the solution to achieve this, is the allocation of water resources within and between economic sectors. To do this, researchers and policymakers need to look at water use amounts, as water impact quantities cannot be allocated. It is clear that different food products within a SFS have different unit WFs. Generally crop products have lower WF amounts than animal products, although there are some exceptions such as nuts (Mekonnen and Hoekstra, 2012; Mekonnen and Hoekstra, 2011). Reducing water stress therefore includes the need for water allocation strategies, such as changing a crop on a farm to a different crop with a lower WF. The same is true in the choice of renewables as a substitute for fossil fuels, for the decarbonisation of the energy sector (Gerbens-Leenes et al., 2009; Holmatov and Hoekstra, 2020; Holmatov et al., 2019; Mekonnen et al., 2015). Apart from intra-sectoral water allocation, inter-sectoral water allocation is also necessary to alleviate water stress, including through river basin transboundary (intra-and international) cooperation (Farinosi et al., 2018; Kapetas et al., 2019; Laghari et al., 2012; Vanham, 2012). Such sectors include agriculture, energy production, industry, municipal water supply and tourism. Much relevant literature on water allocation exists (Davis et al., 2017; Van Aken et al., 2009; Zeitoun et al., 2012).

### 3.4 A fair share of water use for countries or citizens

In the sum of the total products consumed, we have to stay within the environmental boundaries. Vanham et al. (2019b) quantify the WF of consumption of the EU to be 5011 l per person per day, of which a substantial part comes through import from other countries. The food sector account for the largest part (3687 l per person per day), followed by the energy sector (1301 l per person per day). However, a question remains which should be the actual fair share related to EU consumption of the planetary boundary for water. Setting concrete pressure fair share amounts is crucial in order to know how large the reduction should minimally be. Such amounts can be set on a global level up to individual citizens.

### 3.5 Water use assessments require less data, whereas water stress assessments have more uncertainty

For calculating the impact water stress, first the pressure water use is required, and then another layer (water availability) is added. Water availability within a location is generally obtained by means of modelling, sometimes combined with remote sensing, as actual water flow measurements are only conducted in selected locations. Water availability assessments require data/models with all assumptions attached. Water stress assessments therefore have naturally more uncertainty than water use assessments.

In praxis, this means that computing the water use of a farm has less uncertainty than the water stress of a farm. This is relevant information for a policy maker.

### 3.6 Environmental pressures and impacts to communicate to citizens, including for food packaging labelling

The success of some environmental footprints such as the carbon, water and land footprints, is partly due to the fact that they provide a strong communication tool to citizens on how to reduce their pressure on the environment (Vanham et al., 2018a). As dietary and food waste behaviour are key to achieve a SFS (Willett et al., 2019), these communication tools are of high value as they create incentives for such behavioural change. People generally understand their reduction in total diet carbon footprint (Kim et al., 2019), water footprint (Vanham et al., 2018a) or land footprint (Springmann et al., 2018) when shifting from a western to a healthy diet.

**Fig. 4:**
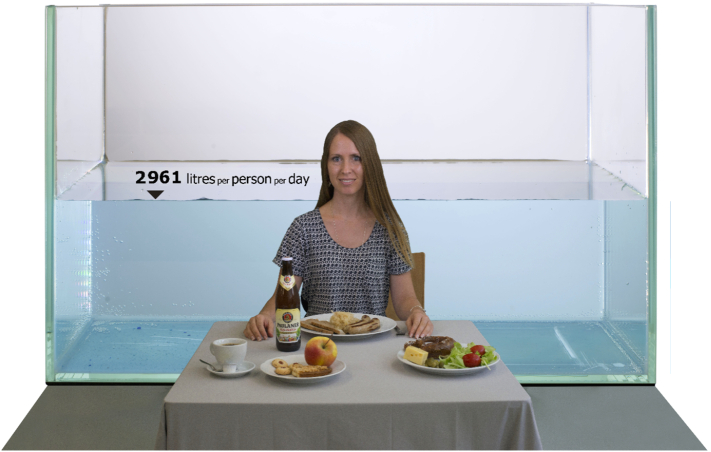
JRC Art and Science Project “The water we eat” is a photographic project displaying the water footprint of the average diet in selected countries, in this case Germany. The project was put on display in museums such as the Museum of Science and Technology Leonardo da Vinci in Milan and the Palace of culture in Sofia. With multiple 10 thousands of visitors, a large public exposure was obtained. Such science-art projects are important ways to communicate science to the general public.

Part of the success of the water footprint in the media and amongst the general public is that water is probably the most recognizable of our resources—anyone knows how much a liter of water is. It is much easier to visualize and understand a bathtub full of water required to produce a piece of bread than more abstract water stress indicators. Different institutions including the European Commission (EC) have recognized this potential, and have used total diet footprints as communication tool such as in the *Urban Water Atlas for Europe* (Gawlik et al., 2017) or the *Future of Cities* report (Vandecasteele et al., 2019). The EC has also initiated relevant science-art projects (Fig. 4), as art-science collaborations are becoming more frequent and recognized to communicate to different audiences (Brennan, 2018; Eldred, 2016).

Food labelling is an important tool to inform citizens on the environmental consequences of their food choice decisions. Which information to use for food packaging should be very carefully and critically assessed. An overview on labelling for different environmental footprints is given by Leach et al. (2016). As argued before, for water, both the amount of water used per unit, as well as the location, linking to the impact, are relevant.

A WF food label can enable to 1) compare a product with other food products; 2) evaluate how a product performs as compared to the benchmark of the same product; and/or 3) relate the product to a “fair share” of available water.

Table 1 provides a theoretical example comparing different indicators for irrigated maize (blue WF, WS, the scarcity-weighted WF) and two other irrigated crops (X,Y), representing a theoretical choice of a consumer between three products. The scarcity-weighted WF has been developed by the LCA-community, using WS as characterization factor, by multiplying the WF with WS (Hoekstra, 2016). It aims to capture both water use and water stress in one indicator, and is e.g. used by Poore and Nemecek (2018) to quantify the impact of different food products. It is measured in quantities of water equivalents (not real water, and should not be communicated as such). Hoekstra (2016) provided critical concerns on this indicator. In our example, the optimal choice based on one of these indicators is when a product has a lower value. Based on the blue WF, a consumer would be incentivized to choose crop Y, then maize and then crop X. Based on WS, the choice would be first crop X, then maize and then crop Y. Based on the scarcity weighted WF, the choice would be first maize, then Y and then X. The three indicators provide totally different preferences. This was already shown for maize regarding WF and WS, as correlation is weak (Fig. 2). The water scarcity-weighted WF aims to quantify impact, and identifies crop X as the least preferable option. This conflicts with the indicator WS, which identifies crop Y as the choice with the highest impact (average WS 4.4). Regarding WS, crop X actually seems the best choice (average WS 1.7). By combining water use and water stress into one indicator, the possibility to compare water use to a benchmark is lost. Using this approach for e.g. food labelling would hereby not grasp information on a product's water use relation to an agreed benchmark. We therefore recommend to use the two first indicators complementary, as discussed in the following outlook section.

**Table 1:**
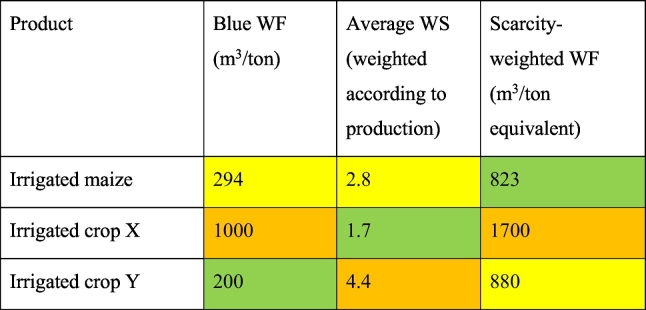
The use of different pressure and impact indicators (the blue WF, WS, the scarcity-weighted WF) can give different recommendations on which product (irrigated maize, crop X or Y) to choose. Colours indicate optimal choice, i.e. the lowest value. Green refers to first choice, yellow to second choice and orange to third or last choice.

## 4 Outlook: SFS water quantity sustainability scheme

**Fig. 5:**
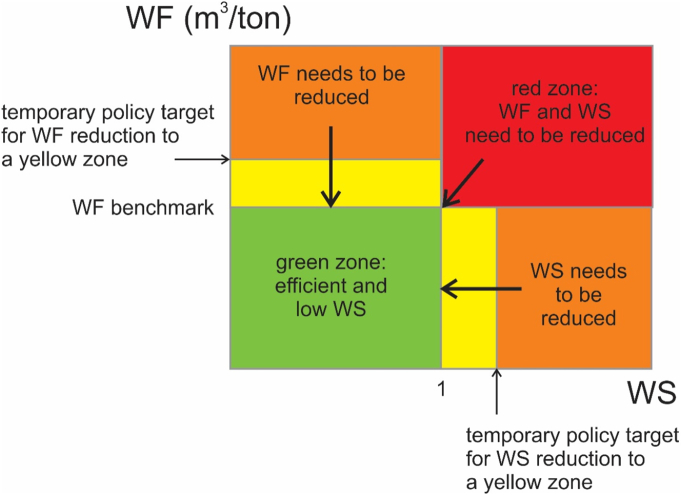
SFS water quantity sustainability scheme (WF = Water footprint; WS = Water stress). The WF includes blue and green water. For purely rainfed products, WS is set at zero. (For interpretation of the references to colour in this figure legend, the reader is referred to the web version of this article.)

We present a SFS water quantity sustainability scheme in Fig. 5. This scheme is divided in four colours: green, yellow, orange and red. The green zone represents agricultural products that have a unit WF up to benchmark amounts and are produced under low water WS. This is the sustainable zone with respect to water quantity. Within the orange zones, either WF amounts need to be reduced to the benchmark value or WS to the low WS zone. The yellow zone presents an intermediate zone, which can politically be set as temporary target within a certain time period. Within the SDG framework, target 6.4 aims to substantially reduce the number of people suffering from water scarcity by 2030. However, a specific quantitative amount is not set. Within the red zone, both the WF and WS are too high, and need to be reduced. This scheme could e.g. also be used for food product package labelling, especially by displaying the colours in a similar way like the Nutri-score (Chantal et al., 2017). When, as discussed earlier, data limitations do not permit to compute water stress, only the WF axis can be used.

This scheme has similarities with the two SDG indicators for SDG target 6.4, i.e. indicator 6.4.1 on water efficiency and indicator 6.4.2 on water stress.

## 5 Conclusions

Environmental pressure indicators inform users on the pressure human activities place on ecosystems rather than on the potential consequences (impact). The overall aim of a SFS is to have a positive or neutral impact on the natural environment. To achieve this goal in the context of global sustainability, there is the need to reduce unsustainable impacts where they already occur, but also to increase efficiencies.

We illustrated this with the example of the water footprint and its related impact indicator water stress. We display, by means of the global water footprint of production of irrigated maize, that there is a weak correlation between water use and water stress, meaning that addressing one of the two indicators does not automatically capture the other. With the same example, we show that unit WF amounts exceeding WF benchmarks occur within low to high impact situations. As the global pool of available water resources is limited, an important component of the solution to overexploitation of freshwater resources in water-stressed regions is to increase water productivity in water-abundant regions. Policies need therefore not only reducing situations of high impact but also increasing the overall water use efficiency or productivity. Product WF benchmarks can help address this.

Another important reason is that the WF has shown great success in its communication to citizens. People understand what a water volume is, because they are confronted with it in their daily lives. The databases behind these numbers are of high quality with detailed spatial resolution. WS data generally are more uncertain, as they require additional information on water availability.

We present a SFS water quantity sustainability scheme, which addresses both WS and WF unit amounts, and uses colour schemes for easy communication. This scheme could be considered as a basis for food labelling. An attempt to combine these two indicators into one looses crucial information on benchmarks.

A recent FAO document on SFSs defined sustainable diets as dietary patterns that have low environmental pressure and impact. All policies on SFSs should do so.

## CRediT authorship contribution statement

**Davy Vanham:** Conceptualization, Formal analysis, Visualization, Writing - original draft. **Adrian Leip:** Conceptualization, Writing - review & editing.

## Declaration of competing interest

The authors declare that they have no known competing financial interests or personal relationships that could have appeared to influence the work reported in this paper.
